# O Isolamento da Parede Posterior na Ablação por Cateter de Fibrilação Atrial Persistente Muda os Desfechos Clínicos?

**DOI:** 10.36660/abc.20240815

**Published:** 2025-02-07

**Affiliations:** 1 Hospital Pró-Cardíaco Serviço de Arritmia Invasiva e Estimulação Cardíaca Artificial Rio de Janeiro RJ Brasil Hospital Pró-Cardíaco – Serviço de Arritmia Invasiva e Estimulação Cardíaca Artificial – Centro de Fibrilação Atrial, Rio de Janeiro, RJ – Brasil

**Keywords:** Fibrilação Atrial, Ablação por Cateter, Eletrocardiografia

A fibrilação atrial (FA) é indiscutivelmente a arritmia mais comum na prática clínica, caracterizada por uma atividade elétrica atrial caótica e rápida com consequente perda da contração atrial e suas graves consequências clínicas já amplamente conhecidas. O diagnóstico é eletrocardiográfico e tem diversas formas de apresentação, sendo a forma persistente, classicamente, a que dura mais de 7 dias e menos de um ano. A forma persistente precoce é quando a duração é superior a 7 dias, porém, inferior a 3 meses e, a forma persistente de longa duração, quando já persiste mais de um ano.^1^ A mudança progressiva da forma paroxística para forma persistente da arritmia varia de 8 a 36% dependendo do tempo de observação da coorte e sempre associada a piores desfechos clínicos.^2,3^

O isolamento elétrico das veias pulmonares, independentemente da fonte de energia utilizada, se tornou a estratégia invasiva padrão-ouro para o tratamento da FA em todas as suas formas de apresentação, com eficácia e resultados amplamente comprovados e baseados na fisiopatologia da arritmia.^4^

A medida que a FA evolui para a forma persistente e subsequente remodelamento elétrico e anatômico, parece bastante razoável oferecer uma estratégia invasiva adicional na abordagem destes pacientes e, neste cenário, o isolamento da parede posterior surgiu como uma proposta coerente. O racional desta estratégia seria o entendimento de que a parede posterior apresenta a mesma origem embriológica que as veias pulmonares e, portanto, com propriedades arritmogênicas similares,^5^ além de muitos plexos ganglionares encontrados nesta região.^6^

Desta forma, o questionamento sobre a real aplicabilidade clínica desta medida estratégica invasiva vem sendo questionada há muito tempo e os achados da literatura são bastante controversos.

Nesta edição da ABC Cardiol, Novaes et al.^7^ apresentaram uma elegante revisão sistemática sobre a eficácia e segurança do isolamento adjuvante da parede posterior em pacientes com FA persistente. Esta revisão incluiu oito estudos e selecionou 1119 pacientes, dos quais 561 (50,1%) se submeteram a isolamento de veias pulmonares (IVP) + isolamento de parede posterior (IPP). Os critérios de inclusão para a seleção dos estudos foram: estudos clínicos randomizados; estudos comparando ablação por cateter envolvendo IVP e IPP versus ablação por cateter com IVP somente; pacientes que se submeteram ao procedimento de ablação para FA persistente; estudos com duração de seguimento de pelo menos 12 meses; e publicações relatando pelo menos um dos desfechos clínicos de interesse. Os desfechos observados foram: recorrência de FA; recorrência de arritmias atriais, isto é, FA, taquicardia atrial, ou *flutter* atrial); complicações clínicas importantes (isto é, derrame ou tamponamento pericárdico; disfunção do nó sinusal ou fístula atrioesofágica); e tempo médio de ablação. Os autores, então, concluíram que o IPP adjuvante parece efetivo em melhorar a FA recorrente, mas não a recorrência de todas as arritmias atriais. O tempo de procedimento foi mais longo com IVP + IPP sem mudança significativa na segurança global e se enfatizou a necessidade de mais estudos para investigar os benefícios em longo prazo.

Recentemente, Kueffer et al.^8^ descreveram os resultados de uma série de 215 pacientes portadores de FA recorrente submetidos ao isolamento elétrico da parede posterior utilizando cateter *pentaspline* PFA. A idade média foi de 70 anos com 70% dos indivíduos do sexo masculino. O procedimento foi completado com sucesso em 100% dos casos com uma média de 36 aplicações de PFA/ paciente. O resultado livre de arritmia em 12 meses foi de 53% (análise Kaplan-Meier) e em 26 pacientes (12%) foi necessário um segundo procedimento em um tempo médio de 6,9 meses. Neste grupo, foi observado IPP consistente em 85% dos casos (22 pacientes). Entre os 4 pacientes com reconexão da parede posterior, 3 apresentavam taquicardia atrial teto-dependente. Os autores então concluíram que o IPP utilizando o cateter *pentaspline* PFA é eficiente e seguro com elevados índices de durabilidade da lesão observados nos procedimentos repetidos e subsequentes.^8^

Uma revisão sistemática e metanálise publicada neste ano envolveu 16 estudos (7 randomizados, 3 prospectivos e 6 retrospectivos) a respeito do valor do IPP nos resultados clínicos em pacientes submetidos a ablação por cateter de FA. Foram incluídos 3.340 pacientes, a maioria de casos de FA persistente e com um tempo médio de acompanhamento de 16,56 meses (1550 IVP + IPP x 1.790 IVP apenas). Os autores concluíram que o IPP se associou a uma diminuição da ocorrência de FA e arritmias atriais sem um aumento da taxa de complicações, especialmente no grupo de FA persistente, e que a crioablação se mostrou mais adequada que a radiofrequência para esta estratégia, enfatizando a necessidade de novos estudos randomizados.^9^

O estudo CAPLA foi um ensaio multicêntrico em que 338 pacientes foram randomizados 1:1 quanto a estratégia de ablação (168 – IVP apenas x 170 – IVP + IPP). O objetivo primário foi a ocorrência de qualquer taquiarritmia documentada com duração superior a 30 segundos após um único procedimento de ablação. A idade média foi de 65,6 anos sendo 76,9% do sexo masculino. Após 12 meses de seguimento, 53,6% do grupo de IVP estavam livres de arritmia e no grupo de IVP + IPP foi observado uma taxa livre de eventos de 52,4%. Os autores então concluíram que em pacientes submetidos ao primeiro procedimento de ablação de FA persistente, o IPP não aumentou de forma significativa a taxa livre de eventos arrítmicos em 12 meses quando comparado a estratégia de IVP apenas e, sugerem que não há evidência suficiente para adicionar empiricamente o IPP como parte da abordagem invasiva desta população.^10^

De forma similar, Ishimura et al. mostraram uma série de 413 pacientes submetidos a ablação de FA onde foram avaliadas a eficácia e durabilidade do IPP com a infusão de etanol na veia de Marshall e concluíram que apesar desta estratégia reduzir o número de ablações extensas da PP, não houve uma melhora da durabilidade do IPP e resultados clínicos.^11^

As explicações para estas disparidades de resultados observados são múltiplas e basicamente relacionadas a dificuldade em criar lesões transmurais e permanentes além da utilização de potências mais baixas de aplicação de radiofrequência pela proximidade com o esôfago.

A resposta precisa e necessária sobre se o IPP muda o desfecho clínico em ablação de FA persistente ainda permanece incerta e aguardando o resultado de outros ensaios clínicos randomizados. No último consenso 2024 EHRA/HRS/APHRS/LAHRS de ablação por cateter e cirúrgica de FA é descrito que 31,6% dos pesquisadores envolvidos realizam o IPP no primeiro procedimento de ablação de FA persistente e 65,8% deles realizam em um segundo procedimento.^12^

Apesar da falta de consenso sobre o assunto e para tomada de decisão pessoal do operador, é sempre válido escutar a opinião dos *experts*, que indiscutivelmente servem como um referencial para uma boa prática médica.
