# Supplementary material for: O Isolamento da Parede Posterior na Ablação por Cateter de Fibrilação Atrial Persistente Muda os Desfechos Clínicos?
Source: Arq Bras Cardiol. 2025 Feb 7;122(1):e20240815. [Article in Portuguese] doi: 10.36660/abc.20240815 (PMC11870122; doi:10.36660/abc.20240815)
Supplement: Translation

# Translation: Does Posterior Wall Isolation in Catheter Ablation of Persistent Atrial Fibrillation Change Clinical Outcomes?

**DOI:** 10.36660/abc.20240815i

**Affiliations:** 1 Rio de Janeiro RJ Brazil Hospital Pró-Cardíaco – Serviço de Arritmia Invasiva e Estimulação Cardíaca Artificial – Centro de Fibrilação Atrial, Rio de Janeiro, RJ – Brazil

**Keywords:** Atrial Fibrillation, Catheter Ablation, Electrocardiography

Atrial fibrillation (AF) is arguably the most common arrhythmia in clinical practice, characterized by chaotic and rapid atrial electrical activity with consequent loss of atrial contraction and its serious clinical consequences, which are already widely known. The diagnosis is electrocardiographic and has several forms of presentation, with the persistent form, being classically, that which lasts more than 7 days and less than a year. The early persistent form is when the duration is more than 7 days but less than 3 months, and the long-term persistent form is when it has persisted for more than a year.^1^ The progressive change from the paroxysmal to the persistent form of the arrhythmia varies from 8 to 36% depending on the observation time of the cohort and is always associated with worse clinical outcomes.^2,3^

Electrical isolation of the pulmonary veins, regardless of the energy source used, has become the gold standard invasive strategy for the treatment of AF in all its forms of presentation, with widely proven efficacy and results based on the pathophysiology of the arrhythmia.^4^

As AF evolves into a persistent form and subsequent electrical and anatomical remodeling, it seems quite reasonable to offer an additional invasive strategy in the approach to these patients and, in this scenario, isolation of the posterior wall has emerged as a coherent proposal. The rationale for this strategy would be the understanding that the posterior wall has the same embryological origin as the pulmonary veins and, therefore, similar arrhythmogenic properties,^5^ in addition to many ganglionic plexuses found in this region.^6^

Thus, the question about the real clinical applicability of this invasive strategic measure has been questioned for a long time and the findings in the literature are quite controversial.

In this issue of ABC Cardiol, Novaes et al.^7^ presented an elegant systematic review of the efficacy and safety of adjunctive posterior wall isolation in patients with persistent AF. This review included eight studies and selected 1119 patients, of which 561 (50.1%) underwent pulmonary vein isolation (PVI) + posterior wall isolation (PWI). The inclusion criteria for study selection were randomized clinical trials; studies comparing catheter ablation involving PVI and PWI versus catheter ablation with PVI alone; patients who underwent the ablation procedure for persistent AF; studies with a follow-up duration of at least 12 months; and publications reporting at least one of the clinical outcomes of interest. The outcomes observed were: recurrence of AF; recurrence of atrial arrhythmias, i.e., AF, atrial tachycardia, or atrial flutter); major clinical complications (i.e., pericardial effusion or tamponade; sinus node dysfunction or atrioesophageal fistula); and mean ablation time. The authors, therefore, concluded that adjuvant PWI appears effective in improving recurrent AF but not the recurrence of all atrial arrhythmias. Procedure time was longer with PVI + PWI without significant change in overall safety and emphasized the need for further studies to investigate long-term benefits.

Recently, Kueffer et al.^8^ described the results of a series of 215 patients with recurrent AF who underwent electrical isolation of the posterior wall using a pentaspline PFA catheter. The mean age was 70 years, with 70% of the individuals being male. The procedure was successfully completed in 100% of cases, with an average of 36 PFA applications/patient. The arrhythmia-free outcome at 12 months was 53% (Kaplan-Meier analysis), and a second procedure was required in 26 patients (12%) at an average time of 6.9 months. In this group, consistent PWI was observed in 85% of cases (22 patients). Among the 4 patients with posterior wall reconnection, 3 had ceiling-dependent atrial tachycardia. The authors concluded that PWI using the pentaspline PFA catheter is efficient and safe, with high rates of lesion durability observed in repeated and subsequent procedures.^8^

A systematic review and meta-analysis published this year involved 16 studies (7 randomized, 3 prospective, and 6 retrospective) regarding the value of PWI in clinical outcomes in patients undergoing catheter ablation for AF. A total of 3,340 patients were included, most of whom had persistent AF and a mean follow-up time of 16.56 months (1550 PWI + PWI x 1790 PWI only). The authors concluded that PWI was associated with a decrease in the occurrence of AF and atrial arrhythmias without an increase in the rate of complications, especially in the persistent AF group, and that cryoablation was more suitable than radiofrequency for this strategy, emphasizing the need for further randomized studies.^9^

The CAPLA study was a multicenter trial in which 338 patients were randomized 1:1 to the ablation strategy (168 – PVI only vs. 170 – PVI + PWI). The primary endpoint was the occurrence of any documented tachyarrhythmia lasting more than 30 seconds after a single ablation procedure. The mean age was 65.6 years, and 76.9% were male. After 12 months of follow-up, 53.6% of the PVI group were arrhythmia-free, and in the PVI + PWI group, an event-free rate of 52.4% was observed. The authors then concluded that in patients undergoing the first ablation procedure for persistent AF, PWI did not significantly increase the arrhythmic event-free rate at 12 months when compared to the PVI-only strategy, and suggest that there is insufficient evidence to empirically add PWI as part of the invasive approach in this population.^10^

Similarly, Ishimura et al. showed a series of 413 patients undergoing AF ablation where the efficacy and durability of PWI with ethanol infusion into the Marshall vein were evaluated and concluded that although this strategy reduced the number of extensive PW ablations, there was no improvement in PWI durability and clinical results.^11^

The explanations for these disparities in observed results are multiple and basically related to the difficulty in creating transmural and permanent lesions in addition to the use of lower powers of radiofrequency application due to the proximity to the esophagus.

The precise and necessary answer as to whether PWI changes clinical outcomes in persistent AF ablation remains uncertain and awaits the results of other randomized clinical trials. In the latest 2024 EHRA/HRS/APHRS/LAHRS consensus on catheter and surgical ablation of AF, it is reported that 31.6% of the researchers involved perform PWI in the first persistent AF ablation procedure, and 65.8% of them perform it in a second procedure.^12^

Despite the lack of consensus on the subject and for the operator's decision-making, it is always worth listening to the opinion of experts, who undoubtedly serve as a reference for good medical practice.
